# Experience-Independent Development of the Hamster Circadian Visual System

**DOI:** 10.1371/journal.pone.0016048

**Published:** 2011-04-27

**Authors:** August Kampf-Lassin, Jenny Wei, Jerome Galang, Brian J. Prendergast

**Affiliations:** 1 Department of Psychology, The University of Chicago, Chicago, Illinois, United States of America; 2 Committee on Neurobiology, The University of Chicago, Chicago, Illinois, United States of America; Vanderbilt University, United States of America

## Abstract

Experience-dependent functional plasticity is a hallmark of the primary visual system, but it is not known if analogous mechanisms govern development of the circadian visual system. Here we investigated molecular, anatomical, and behavioral consequences of complete monocular light deprivation during extended intervals of postnatal development in Syrian hamsters. Hamsters were raised in constant darkness and opaque contact lenses were applied shortly after eye opening and prior to the introduction of a light-dark cycle. In adulthood, previously-occluded eyes were challenged with visual stimuli. Whereas image-formation and motion-detection were markedly impaired by monocular occlusion, neither entrainment to a light-dark cycle, nor phase-resetting responses to shifts in the light-dark cycle were affected by prior monocular deprivation. Cholera toxin-b subunit fluorescent tract-tracing revealed that in monocularly-deprived hamsters the density of fibers projecting from the retina to the suprachiasmatic nucleus (SCN) was comparable regardless of whether such fibers originated from occluded or exposed eyes. In addition, long-term monocular deprivation did not attenuate light-induced c-Fos expression in the SCN. Thus, in contrast to the thalamocortical projections of the primary visual system, retinohypothalamic projections terminating in the SCN develop into normal adult patterns and mediate circadian responses to light largely independent of light experience during development. The data identify a categorical difference in the requirement for light input during postnatal development between circadian and non-circadian visual systems.

## Introduction

Organisms coordinate physiology and behavior to anticipate daily changes in the environment. The suprachiasmatic nucleus (SCN) of the hypothalamus acts as the master circadian pacemaker for physiological and behavioral circadian rhythms [1], coordinating rhythmicity among central and peripheral oscillators [2]. The SCN maintains an endogenous periodicity of approximately 24 h absent any environmental input, but is reset in the presence of periodic time cues (“Zeitgebers”) in the environment, a process termed entrainment. Among the majority of vertebrates, the most potent Zeitgeber for entrainment of the SCN is the daily light-dark cycle.

Light is communicated from the retina directly to the SCN through a photoreceptive pathway distinct from that of the retinothalamocoritcal primary visual system [3] by melanopsin-containing retinal ganglion cells (RGCs) [4], [5], which project via the retinohypothalamic tract (RHT), and terminate in the SCN [3]. Illumination level is the parameter of light input most important in stimulating the RHT and in driving SCN phase-resetting responses [6]. This stands in contrast to the primary visual system, comprised of the polysynaptic projection from retina to thalamus to visual cortex, which processes patterned input consisting of color, contour and motion, but is relatively poorer at perceiving changes in brightness [7]. The RHT and SCN thus constitute a distinct circadian visual system [8], which is sufficient [9] and necessary [10], [11] for circadian entrainment to light.

The capacity for sensory experience during development to alter neural connectivity and function is a common feature of sensory systems [12]. Neural connections are guided initially by neurotrophic factors and spontaneous patterns of activation that occur before the onset of sensory experience [13], but the fine-tuning and maintenance of these circuits proceeds according to Hebbian principles of synaptic plasticity [12], [14]. Canonical examples of this phenomenon are evident in the primary visual system: in adulthood, animals deprived of patterned visual input in one eye during a period of early postnatal development exhibit loss of function among cortical circuits normally driven by the deprived eye. Visual cortex in such animals becomes dominated by input from the exposed eye [15], [16]. This experience-dependent developmental plasticity has likewise been demonstrated in somatosensory [17] and auditory cortex [18] following unilateral deprivation of the respective sensory input.

It has been well established that exposure to a circadian Zeitgeber (e.g. a light-dark cycle) during postnatal development is not necessary for the emergence of circadian rhythms in physiology and behavior (e.g., core body temperature [19]; plasma corticosterone [20]; locomotor activity [21]). The maternal circadian clock entrains the fetal SCN either during gestation [22], [23], [24], during early postnatal development [25], or both [26], depending on the species. Within-litter circadian synchrony is abolished in pups of SCN-lesioned dams, but individual pups still express normal circadian rhythms [26], [27]. Maternal rhythms are conferred upon fetuses during gestation, and cross-fostering experiments show that pups do not adopt the phase of a foster dam under constant darkness [28], [29]. Whereas the development of circadian locomotor activity rhythms absent any history of photic input demonstrates that light exposure is not necessary for the development of circadian pacemaker, it is unclear whether the mechanisms that govern photic resetting of the pacemaker by light input develop in a similar experience-independent manner.

In contrast to the geniculocortical pathways of the primary visual system, little is known about whether functional connectivity of the RHT projecting from either retina is dependent on functional transmission of light information from the retina to the SCN during development. Studies of RHT development identified minor deficits in re-entrainment following unilateral enucleation [30]; however, this analysis could only be used to evaluate the function of projections from the intact, light-experienced eye.

The aim of these experiments was therefore to determine whether use-dependent plasticity governs development and maintenance of this sensory pathway. A reversible monocular deprivation manipulation was implemented, which was sufficient to block functional light signals from reaching the targeted retina. Following many weeks of such unilateral monocular deprivation, we evaluated whether a previously-occluded eye could mediate entrainment, light-induced phase shifts, and light-induced immediate-early gene expression in the SCN. In a parallel experiment, cholera-toxin fluorescent tract-tracing was used to evaluate effects of monocular deprivation on the development of SCN innervation by the RHT.

## Results

### Experiment 1: Verification of deprivation manipulation

**Figure 1:**
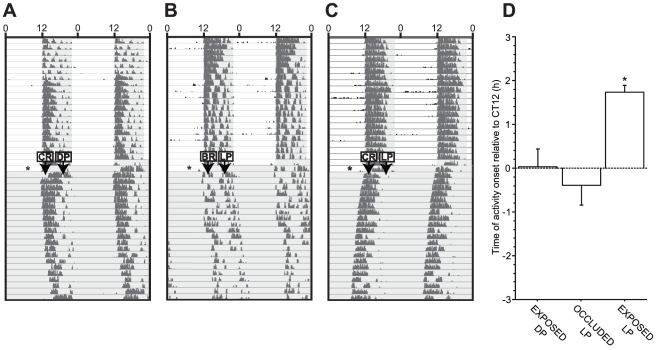
Occlusion of circadian light perception. Representative double-plotted locomotor activity records of Syrian hamsters in experiment 1. Clock time is indicated on the horizontal axis along the top of each actogram. Lights on/off are indicated by light and shaded areas of the actogram. Hamsters were blinded in the left eye via optic nerve transection (indicated by *), transferred to DD, and subjected at ZT19 to ***A*** a control dark pulse (DP) with a clear lens over the right eye (CR), ***B*** a 15 min light pulse (LP) with an occluder over the right eye (BR), or ***C*** a 15 min LP with a clear lens over the intact eye. ***D*** Mean (± s.e.m.) magnitude of the phase shift in activity onset as measured 5 days later. * p<0.001 vs. all other groups.

To evaluate the efficacy of lens occluders, and to determine whether light entering through the eye contralateral to the occluder stimulates the retina of the occluded eye, unilaterally-denervated hamsters were exposed to light pulses in the presence or absence or an occluder in the non-denervated eye. Among hamsters with transections of the left optic nerve (ONx), application of an opaque occluder lens to the right eye abolished the phase shifting response to a 15 min light pulse (LP) at ZT19 (Zeitgeber Time 19; 7 hours after lights off) (F_2, 11_ = 10.699, p<0.005, Fig. 1). Hamsters bearing a clear lens over the right eye exhibited an approximately 2 h phase advance 5 days following the LP. In contrast, hamsters subjected to a LP after being fitted with an occluder over the right eye did not exhibit phase advances, nor did hamsters receiving the DP. These later groups exhibited free-running activity in continuous darkness (DD). 6 hamsters that received ON transections in both eyes exhibited free-running circadian locomotor activity rhythms with periods (τ) = 24.08±0.21 SEM (≥24.20 h or ≤23.56 h) under a 24 h light-dark cycle (τ ≠ 24, t_5_ = 6.951, p<0.001), whereas 6 hamsters that received a sham operation in both eyes maintained entrainment with τ = 23.99±0.01 SEM (≥23.96 h and ≤24.02 h), not differing from 24 h (t_5_ = −0.955, p = 0.383).

### Experiment 2: Circadian effects of monocular deprivation

**Figure 2:**
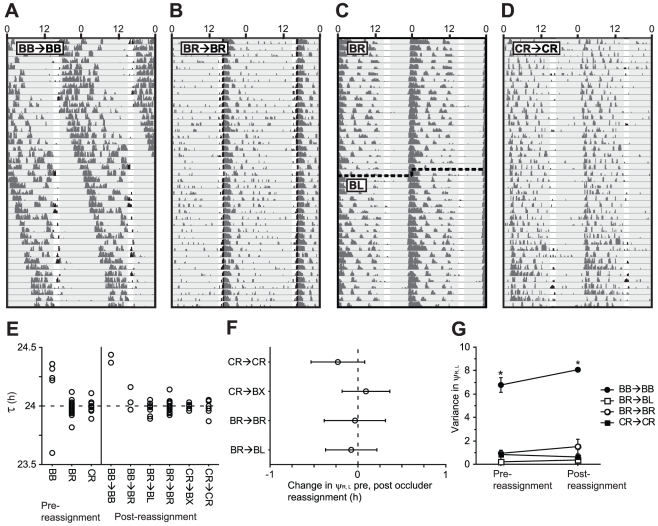
Stability of entrainment following occluder reassignment. Distribution of periods (τ) of the circadian locomotor activity rhythm in experiment 2 over the 3 weeks prior to and 3 weeks following occluder reassignment on week 10. ***A***–***D*** Representative double-plotted locomotor activity records of Syrian hamsters bearing: ***A*** occluders in both eyes (BB→BB), ***B*** an occluder in the right eye (BR→BR), ***C*** an occluder that was moved from the right eye to the left on week 10 (BR→BL), and ***D*** a clear lens in the right eye (CR→CR). ***E*** Distribution of τ over the 3 weeks before and 3 weeks after occluder reassignment. ***F*** Mean (± s.e.m.) change in the phase angle of entrainment of the onset of locomotor activity relative to the onset of light (ψ_R, L_) from the 3 weeks before occluder reassignment to the 3 weeks after. CR→BX hamsters received an occluder in the left or right eye after week 10, prior to which they received a clear lens in the right eye. ***G*** Variance in ψ_R, L_ over the 3 weeks before and 3 weeks after occluder reassignment. * p<0.005 vs. all other groups.

Hamsters were subjected to monocular or binocular occlusion via opaque occluders for 10 weeks, after which, in a subset of lensed hamsters, occlusion was performed in the contralateral eye, or not at all (“lens switch”, see *Materials and Methods*). During the three weeks preceding the lens switch (weeks 8–10), BB hamsters exhibited a free-running locomotor activity rhythm (Fig. 2A). Periods of the circadian locomotor activity rhythms (τs) of the 5 hamsters bearing opaque occluder lenses in both eyes (BB) were ≠ 24 h (t_3_ = 8.626, p<0.005). 4 hamsters exhibited τ>24 h, while in the remaining hamster τ = 23.60 (Fig. 2A, 2E). Hamsters bearing an occluder in the right eye (BR, Fig. 2B) and hamsters bearing a clear lens in the right eye (CR, Fig. 2D) exhibited τ values that did not differ from 24 h (BR: t_20_ = −1.606, p = 0.124; CR: t_11_ = −0.109, p = 0.915, Fig. 2E).

Lens reassignment/switch on week 10 (see *Materials and Methods*) or maintenance of lens conditions did not yield significant changes in patterns of entrainment. BB→BB hamsters continued free-running activity rhythms with τ>24.37 h. Among CR→CR and BR→BR hamsters, there were no changes in the period of the locomotor activity (Fig. 2E), phase angle of entrainment (ψ_R, L_; Fig. 2F), or stability of entrainment (Fig. 2G). Finally, among hamsters in which the occluder was moved from the right to the left eye on week 10 (BR→BL), there were no significant changes in τ (Fig. 2E), ψ_R, L_ (Fig. 2F), or stability of ψ_R, L_ (Fig. 2G) during the 3 weeks following lens reassignment.

**Figure 3:**
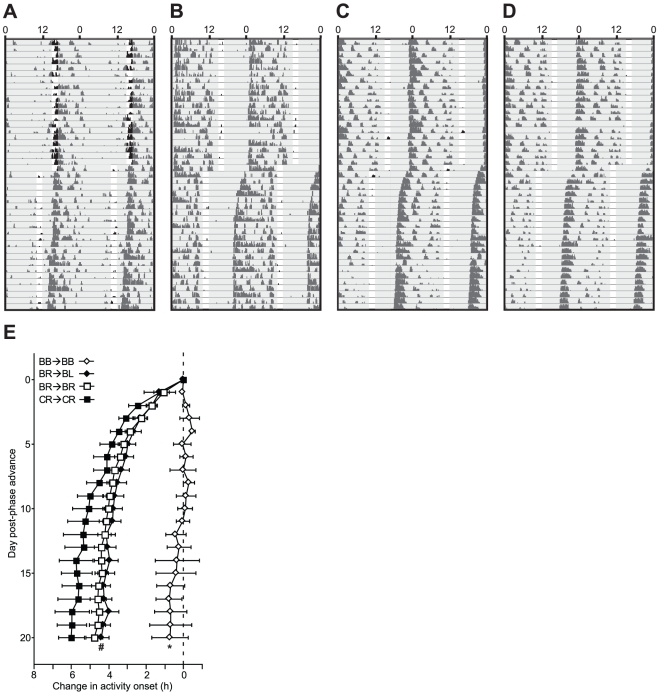
Light-induced phase shifts of the circadian locomotor activity rhythm after occluder reassignment. ***A***–***D*** Representative double-plotted locomotor activity records of Syrian hamsters bearing: ***A*** occluders in both eyes (BB→BB), ***B*** an occluder in the right eye (BR→BR), ***C*** an occluder that was moved from the right eye to the left on week 10 (BR→BL), and ***D*** a clear lens in the right eye (CR→CR). Clock time is indicated on the horizontal axis along the top of each actogram. Lights on/off are indicated by light and shaded areas of the actogram. ***E*** Mean (± s.e.m.) daily locomotor activity onsets of hamsters bearing ocular occluders during the 3 weeks immediately following a 5-hour phase advance of the light-dark cycle. * p<0.05 vs. all other groups, # p<0.05 vs. the CR-CR group.

On week 16, the photocycle was advanced by 5 h in a single day. Hamsters with one or more photorecipient eyes exhibited gradual phase advances during the 3 weeks following the 5 h phase advance (Fig. 3A–D). A main effect of time (F_20, 660_ = 83.410, p<0.0001) and an interaction between time and lens treatment group (F_100, 660_ = 2.087, p<0.0001) was observed on the change in activity onsets from the day prior to the phase shift (Fig. 3E). BB→BB hamsters largely failed to alter their time of activity onset, whereas all other groups accomplished 4–6 h phase advances over this interval (F_20, >40_>2.658, p<0.01, all comparisons); CR→CR hamsters exhibited the largest phase advances (≈6 h), and BR→BL hamsters exhibited the smallest (≈4 h) (F_20, 280_ = 2.413, p<0.001, Fig. 3E). Among BR hamsters, lens reassignment to the contralateral eye (i.e., BR→BL) did not significantly alter the rate or magnitude of reentrainment response to the 5 h phase advance (Fig. 3E).

### Experiment 3: Perceptual effects of monocular deprivation

**Figure 4:**
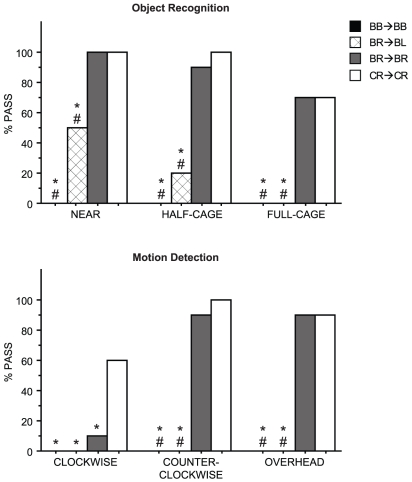
Visual acuity following light occlusion. Proportion of hamsters exhibiting behavioral responses to a sunflower seed 2 cm away (NEAR), one half-cage length away (HALF-CAGE), one full cage length away (FULL-CAGE), rotated clockwise about the head at a distance of 2 cm (CLOCKWISE), rotated counter-clockwise about the head at a distance of 2 cm (COUNTER-CLOCKWISE), and moved from the NEAR position to 2 cm overhead (OVERHEAD). Group abbreviations as in Fig. 3. * p<0.05 vs. CR→CR, # p<0.05 vs. BR→BR.

On week 15, hamsters from experiment 2 were presented visual acuity tasks (see *Materials and Methods*). Hamsters in all treatment groups successfully detected a sunflower seed when presented in contact with the cheek. Visual acuity was impaired in hamsters presented with a seed to a previously-occluded eye. When the seed was presented a half- or full cage-length away, or overhead, BR→BL hamsters passed fewer seed trials relative to both BR→BR and CR→CR hamsters (χ^2^>7.20, df = 1, p<0.05, all comparisons, Fig. 4). Performances were comparable among hamsters with one exposed eye only if that same eye had also been exposed during the first 10 weeks of light exposure (i.e., BR→BR and CR→CR groups; p>0.05, all comparisons). BB→BB hamsters failed all visual acuity tests.

### Experiment 4: Effects of monocular deprivation on light-induced c-fos expression in the SCN

Following 38 weeks of ocular occlusion, hamsters from the preceding experiments were transferred to DD, and 3 weeks later, SCN c-Fos immunoreactivity (Fos-IR) was measured following a brief (1 min), non-saturating light pulse at circadian time 19 (CT19) (for groups and light treatments, see *Materials and Methods*). Across all treatment conditions, number of Fos-IR cells was comparable between the SCN ipsilateral and contralateral to the eye receiving an LP (F_1, 78_ = 0.05, p = 0.946); therefore, cell counts for the left and right SCN were pooled for each hamster. In addition, SCN Fos-IR cell counts were comparable in CR→CR hamsters that received a LP in the left and those that received a LP in the right eye (F_1, 7_ = 0.02, p = 0.968); thus, eyes treated with a clear lens were thus regarded as exposed.

**Figure 5:**
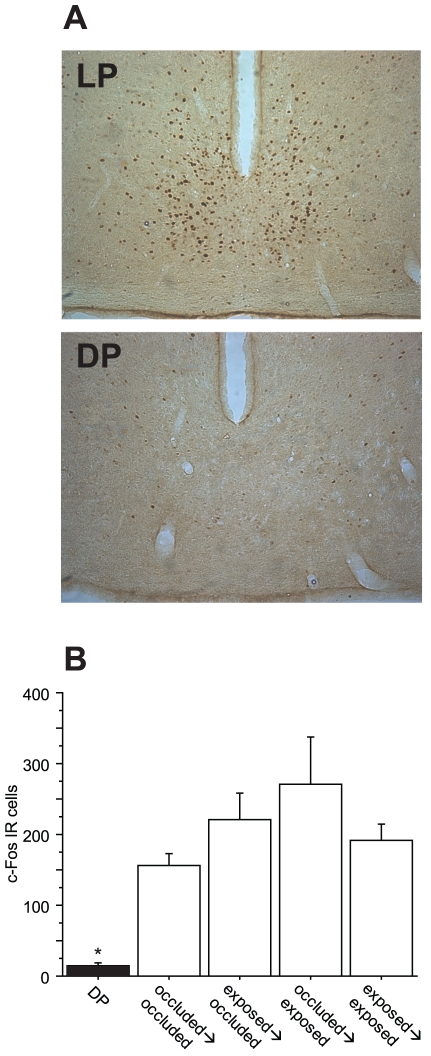
Light-induced c-*fos* expression in the SCN. ***A*** Representative images of DAB-labeled Fos-IR cells in SCN coronal sections from 2 Syrian hamsters. ***B*** Mean (± s.e.m.) number of c-Fos immunoreactive (IR) cells in both SCN 90 minutes after the delivery of a dark pulse (DP) or a light pulse (LP) at CT19 (see methods for details) to an eye that had been either exposed or occluded from weeks 1–10 and from weeks 11–38. * p<0.0001 vs. all other groups.

Hamsters that received a dark pulse (DP) had fewer SCN Fos-IR cells than those that received a LP (F_4, 39_ = 5.474, p<0.005, Fig. 5). There were no significant differences in the number of Fos-IR cells among the four possible treatment histories of the eye that received a LP (occluded→occluded, occluded→exposed, exposed→occluded or exposed→exposed; F_3, 35_ = 1.355, p = 0.273).

### Experiment 5: Effects of monocular deprivation on retinohypothalamic projections

**Figure 6:**
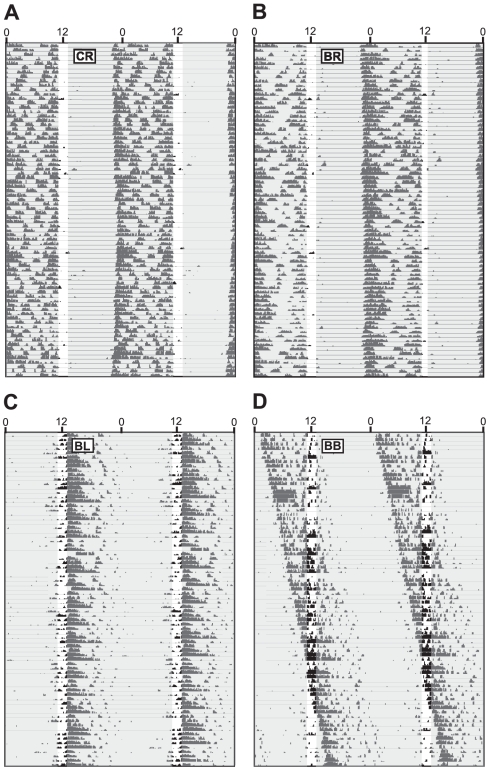
Representative double-plotted locomotor activity records of Syrian hamsters in experiment 5. Clock time is indicated on the horizontal axis along the top of each actogram. Lights on/off are indicated by light and shaded areas of the actogram. Hamsters were subjected to the following occlusion treatments for 38 weeks: ***A*** a clear lens over the right eye (CR), ***B*** an occluder over the right eye (BR), ***C*** an occluder over the left eye (BL), or ***D*** occluders over both eyes (BB).

To determine if long-term monocular occlusion affects development of the RHT, separate groups of hamsters were subjected to eye-specific lensing, or control lensing, for 38 weeks, followed by fluorescent dye-conjugated cholera toxin-b subunit labeling of retinal ganglion cells projecting to the SCN (see *Materials and Methods*). Hamsters in this study exhibited patterns of locomotor activity comparable to those observed prior to the lens switch in experiment 2 (Fig. 6). BR, BL and CR hamsters maintained entrainment to the light-dark cycle for 38 weeks, whereas BB hamsters exhibited free-running locomotor activity rhythms.

**Figure 7:**
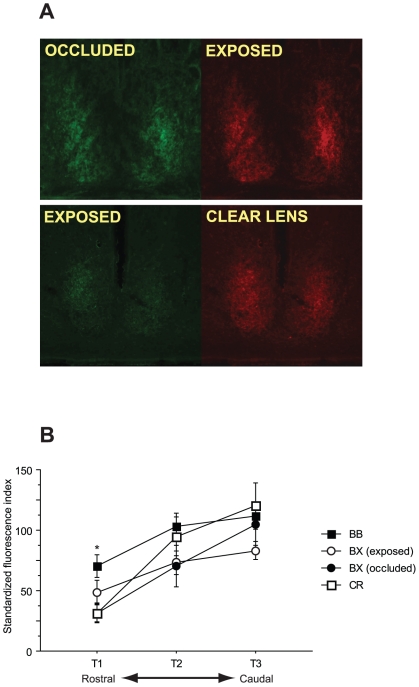
RHT projections following occlusion. ***A*** Representative images of fluorescently-labeled retinal SCN afferents from 2 Syrian hamsters. ***B*** Mean (± s.e.m.) standardized fluorescence index (SFI) of SCN receiving projections from occluded and exposed eyes. Data depict 3 rostral-caudal sections of the SCN (T1, T2, and T3; see Methods) of hamsters bearing ocular occluders for 38 weeks. * p<0.05 vs. both BX (occluded) and CR.

In CR hamsters, there was no difference in projection density from the clear-lens treated eye compared to that from the non-lensed eye (F_1, 13_ = 0.035, p = 0.854; Fig. 7). Both eyes were regarded as exposed in this group. Similarly, there was no difference in projection density between the left and right eyes of BB hamsters. The density of the RHT projection to the SCN from the occluded eye did not differ between the BR and BL groups at any of the 3 serial sections (T1–T3) through the rostrocaudal extent of the SCN (F_1, 10_<2.548, p>0.146), nor did SCN standardized fluorescence index (SFI) from the exposed eye differ between these groups (F_1, 10_<2.743, p>0.129). Accordingly, BR and BL groups were combined into a single monocularly-deprived group, hereafter referred to as “BX.” In total, 4 classifications of eyes were compared in this analysis: CR, BB, BX (occluded), and BX (exposed).

Among BX (i.e., BR and BL groups) hamsters, SFI did not differ between SCN ipsilateral and contralateral to the injected eye in any of the any of the 3 sections (F_1, 10_≤0.680, p≥0.7984). SFI over the bilateral SCN was used in all subsequent comparisons.

There was a main effect of treatment group on SFI in the most rostral (T1) region of the SCN (F_3,46_ = 4.409, p<0.05, Fig. 7), but main effects were not evident in the T2 (F_3,58_ = 2.063, p = 0.115) or T3 (F_3,58_ = 0.704, p = 0.554) sections. SFI was greater in the T1 section of the SCN in BB hamsters relative to BX (exposed) and CR hamsters (p<0.005, all comparisons).

## Discussion

Monocular occlusion treatments sufficient to render an eye incapable of mediating form and motion perception did not affect the ability of that eye to mediate circadian entrainment or circadian phase resetting responses to light. No decrements were evident in a previously-occluded eye's ability to induce SCN c-Fos expression in response to a brief, non-saturating light pulse, and no obvious anatomical changes were evident in the density of the RHT projecting from monocularly occluded eyes to the SCN. These measures do not constitute an exhaustive inventory of circadian function, thus it is possible that monocular occlusion exerted subtle effects that currently remain undetected. However, taken together, the present outcomes provide strong anatomical, behavioral, and physiological evidence that the functional development of retinal inputs to the circadian pacemaker is not dependent on light input during postnatal development.

The present study used ocular occluders that blocked circadian perception of light input from a targeted eye (Expt. 1). All hamsters with at least one non-occluded eye entrained to the light-dark cycle through week 10 (Figs. 2, 3), consistent with reports in rats and hamsters that only one eye is required for entrainment of the circadian system to full photocycles [31], [32]. Following lens reassignment on week 10, BR→BL hamsters maintained entrainment to the light-dark cycle using an eye that was previously occluded, and entrainment mediated via a previously-occluded, light-naïve eye was quantitatively indistinguishable from that mediated by an experienced eye (Fig. 2E–G). In addition, when subsequently challenged with a 5 h advance in the photocycle, BR→BL hamsters re-entrained in a manner that was comparable in rate and magnitude to that exhibited by BR→BR hamsters. CR→CR hamsters exhibited faster and larger phase advances to the 5 h phase shift relative to other groups, however, this is likely due to the two-fold increase in light input to the circadian pacemaker in these animals [31], [32]. Together, the retention of normal entrainment and phase-shifting capabilities in BR→BL hamsters suggests that functional maturation of the sensory systems that mediate light input to the circadian pacemaker is not influenced developmentally by use-dependent plasticity and is not dependent on light input during postnatal development.

The functional integrity of the circadian visual system following this extended interval of monocular deprivation stands in marked contrast to the classical (form, motion perception) visual system. In BR→BL hamsters, early light deprivation induced a profound lack of visual acuity: in the half-cage, full-cage, and overhead trials, BR→BL hamsters were functionally blind compared to BR→BR hamsters (Fig. 4).

Exposure to full (albeit relatively short, 2 h) photoperiods likely provided super-saturating light input to the circadian pacemaker [33]. Such a strong input may have obscured modest decrements in the ability of a previously-occluded eye to communicate light information to the circadian timing system. Therefore we next assessed SCN molecular responses to non-saturating light stimuli. To assess SCN responses to light originating from eyes with known histories of experience, hamsters were challenged with a 1 min, 8.22 µW/cm^2^ light pulse at CT19, a treatment that reliably induce phase advances of the circadian pacemaker and c-Fos expression in the SCN [8], [34]. Upon immunohistochemical examination of SCN c-Fos expression, no relationship was evident between an eye's history of light experience (occluded vs. exposed) and its ability to mediate light-induced c-Fos activation in the SCN (Fig. 5). Indeed, light-information mediated by an eye that had been occluded during a prolonged interval of postnatal development elicited SCN c-Fos expression that was comparable to that mediated by an eye that had never been occluded. These data indicate that, in common with behavioral measures of circadian function, early molecular events in the process of light-induced phase resetting appear impervious to postnatal light experience.

Together, these outcomes suggest that the requirements for light input during development differ markedly for visual processes that mediate image formation and motion perception relative to those that mediate circadian entrainment. Whereas the primary visual system in this and other species is dependent on patterned input for normal functional development ([15], [16], Fig. 4), the circadian visual system appears to have no such requirements. The results thus identify a categorical difference between the experience-dependence of normal adult function in the primary visual system and the experience-independent development of function in the circadian visual system. The ability of monocularly deprived eyes to sustain entrainment, mediate phase resetting responses, and induce molecular events in the SCN is remarkable when compared to the consequences of monocular deprivation on functional connectivity in the primary visual system. In cats, a period of monocular deprivation of only 10 days during early development was sufficient to markedly suppress the ability of the deprived eye to drive activation of neurons in visual cortex [35], and in rats, one month of monocular deprivation during early postnatal development resulted in >90% of cells in a normally binocularly-innervated area of visual cortex being dominated by input from the nondeprived eye [36]. The circadian visual system suffers no such functional effects after substantially longer intervals of deprivation.

Finally, to further investigate the apparent developmental resilience of the circadian visual system, in a final study we quantified the density of RHT projections to the SCN from eyes that were occluded or exposed for 38 weeks of postnatal light experience. In this experiment, retinas of occluded eyes never received a functional light signal, yet, their ganglionic projections to the SCN were retained in adulthood in densities that were indistinguishable from those of binocularly sighted hamsters. Moreover, even within monocularly occluded hamsters, the density of RHT projections originating from occluded eyes was identical to those originating from non-occluded eyes (Fig. 7).

The only morphological effect of occlusion was observed in the most rostral section (T1) of the SCN. Here, hamsters subjected to binocular occlusion (BB) exhibited significantly higher fiber density relative to hamsters that were monocularly deprived (BX) or nondeprived (CR). Binocular deprivation reduces inputs to visual cortex from each eye, but this effect is not as robust as the effects evident following comparable intervals of monocular deprivation [37]. This observation was, therefore, somewhat unexpected. Hamster RHT projections first reach the SCN at postnatal day 0 and are distributed in an adult-like pattern by P10 [38]. SCN innervation by the RHT may be guided by the spontaneous activity of retinal ganglion cells, which also drives retinotopic organization of the thalamic visual nuclei prior to eye opening [13], or may be driven by an activation-independent mechanism. In either case, the complete absence of light input following the instantiation of a photocycle led to a modest preservation of projections in this rostral-most SCN region. Light input to the SCN after P10 may activate mechanisms that prune less-functional RHT inputs (cf. [39]), but if such a mechanism is operant, then the threshold for light input is absolute (i.e., any light input leads to synaptic pruning), and limited to only a small rostral region of the SCN. Moreover, the biological significance of such an anatomical response is unclear, as it does not appear to give rise to differences in the functional capabilities of the circadian visual system in adulthood. In mice, postnatal rearing in DD has been shown to augment the tau-lengthening effect of LL in adulthood [40]. Effects of early light deprivation on the adult circadian system, when evident, appear to be modest. In general, the strong similarity between projections from occluded and exposed eyes of BX hamsters demonstrate that the RHT is anatomically impervious to deprivation during postnatal development.

From a functional perspective, the relative absence of experience-dependent plasticity in the circadian visual system distinguishes this system from the primary visual system; however, these features bear strong similarities to properties of retinal projections to the LGN, which are more anatomically resilient to both binocular and monocular deprivation than are geniculocortical projections [41], [42]. RGCs projecting through the RHT and RGCs that project through the postchiasmatic primary optic tract to the geniculate nuclei thus appear to share this resilient property. Concurrent analyses of both retinohypothalamic and retinogeniculate afferents would afford additional insights into this conjecture; however, the present study did not investigate effects of monocular occlusion on the retinogeniculate projections. Experience-independent functional connectivity may be evident in sensory projections to subcortical structures, whereas experience-dependent plasticity may be largely restricted to thalamocortical pathways.

In summary, the present study demonstrates that in hamsters, postnatal monocular occlusion yields functional blindness in the primary visual system but does not disturb normal development of the circadian visual system. Eyes that, during an extended period of postnatal development, never mediated circadian entrainment, phase-resetting responses to light, or SCN molecular responses to light, were nevertheless capable of doing so in adulthood. Moreover, prolonged monocular occlusion yielded no obvious changes in RHT morphology. Assays of multiple behavioral, physiological, and anatomical consequences of monocular occlusion provide convergent evidence in support of the hypothesis that functional development of the circadian visual system proceeds independent of light input during postnatal development.

## Materials and Methods

### Animals and housing

Male and female Syrian hamsters (*Mesocricetus auratus*) were bred and raised postnatally in continuous darkness (DD) with a dim (<0.1 lux) red overhead light to facilitate animal husbandry. This light remained on at all times in this study. Light of this intensity does not alter patterns of entrainment and does not interfere with light-induced immediate-early gene (c-*fos*) expression in the SCN [34], [43], thus periods of exposure to only this light are referred to as *darkness* throughout the study. At all times, hamsters were housed in polypropylene cages (39.3 cm×28.5 cm×19.4 cm); food and water were available ad libitum, and an ambient temperature of 20±2°C was maintained. At postnatal day 16–21, hamsters were weaned and individually housed in identical cages containing 12 cm stainless steel wire running wheels for the remainder of the study. All animal treatments described in this experiment conformed to the USDA Guidelines for the Care and Use of Laboratory Animals and were approved by the University of Chicago Institutional Animal Care and Use Committee.

### Activity measurements

Locomotor activity of each hamster was monitored via a running wheel fitted with magnetic contacts 180° apart and a magnetic reed switch set to record an event at each half-rotation of the wheel. Events were compiled into 6 min bins and analyzed using ClockLab software (Actimetrics, Evanston, IL). Daily activity onsets were determined automatically by Clocklab, which defines an onset as the time at which there is a maximum difference between the preceding and following 6 h windows in the number of bins in which the number of counts exceeds 20% of all non-zero bins. In a minority of cases, daily onsets were not identifiable automatically and were determined through visual inspection of the actograms by an experimenter who was blind to treatment groups. Circadian parameters were identified according to methods described in Evans et al. [44]. The period of the circadian locomotor activity rhythm (τ) was measured by fitting a regression line to activity onsets over the course of 3 weeks. Phase shifts were determined by calculating the difference between post-pulse activity onset and a regression line projected from pre-pulse activity onsets.

### Occluders

Ocular occluders and clear (control) contact lenses were manufactured through a process identical to that described elsewhere [32], [45]. Briefly, a ball bearing mounted on a drill press was pressed into a heated sheet of black or clear plastic; the plastic sat atop a hemispherical mold with a diameter slightly larger than that of the bearing. The occluder was removed from the mold with a hole punch tool, and its edges were trimmed and polished with fine sandpaper and cotton. A range of occluder sizes was used (4–6 mm diameter) to accommodate the growth of the eye during development. Occluders were hemispherical and completely covered the exposed surface of the eye. These occluders effectively prevent light-induced behavioral phase shifts ([32]; present data) and c-Fos expression in the SCN [32]. When not in use, occluders were stored in contact lens cleaning solution (Multi-Purpose Solution, Walgreens, Deerfield, IL). Occluders were cleaned with sterile cotton swabs daily before use.

### Experiment 1 Procedure: Verification of deprivation manipulation

To evaluate the efficacy of occluders in blocking light-induced phase shifts, and to determine whether light entering through the eye contralateral to the occluder functionally stimulates the RHT projecting from the eye bearing the occluder, unilaterally optically-denervated hamsters were exposed to light pulses in the presence or absence of an occluder on the non-denervated eye. Phase shifts were assessed using an Aschoff Type-II design [46].

Adult hamsters (n = 15) were housed under a 14L∶10D light-dark cycle (lights on at 15:00 CST) in cages equipped with running wheels. During the light phase, hamsters were anesthetized with sodium pentobarbital (Nembutal, 50 mg/kg, i.p.) and the left optic nerve was surgically transected using microscissors (n = 5; ONx), leaving the orbit in place. Buprenorphine (Buprenex, 0.5 mg/kg, s.c.) was administered as postoperative analgesia. Hamsters were transferred to continuous dakness (DD) on the first dark phase after surgery. On the second day of exposure to DD, the right eye was covered with an opaque (black) occluder lens (n = 5; BR) or a control (clear) lens (n = 5; CR). All hamsters then received a light pulse (15 min, 400 lux; LP) at ZT19. Five additional control hamsters received CR treatment but received a control dark pulse (DP) rather than an LP. Phase shifts of the locomotor activity rhythm were quantified as described above based on locomotor activity collected during the following 3 weeks in DD. Additionally, 6 hamsters received the ONx treatment in both eyes, and another 6 hamsters received a sham operation in which each eye was gently handled but the ON not transected. These hamsters were maintained in the 14L∶10D light-dark cycle throughout.

### Experiment 2 Procedure: Circadian effects of monocular deprivation

#### Animals and initial lensing conditions

Male and female Syrian hamsters (n = 42) were housed in DD from ≥3 days prior to birth until 4–5 weeks of age ( = week 0). On week 0, hamsters were randomly assigned to one of several contact lens treatment groups (described below) and transferred to a 2L∶22D light-dark cycle (lights on at 15:00 CST).

Lensing began on the day prior to implementation of the LD cycle, and hamsters received the same lensing treatment every day for the next 10 weeks. One group of hamsters received opaque occluders in both eyes each day (BB; n = 5). Additional groups received an opaque occluder in the right eye (BR; n = 25), or a clear lens in the right eye (CR; n = 12). Each day, hamsters were lightly anesthetized with isoflurane vapors and lenses were placed under the eyelid. Lenses were inserted by holding the hamster firmly by the jaw and retracting the eyelid. The lens was then placed over the eye and the eyelid was allowed to close over the lens. The shape of the lenses provided a snug fit underneath the eyelid, which kept the lens secured over the eye, but allowed the eyelid to close freely. Lenses were removed by retracting the eyelid and pulling the lens off the eye. The application and removal procedures lasted <1 min per animal. Lenses were inserted ≤5 hours before the onset of the light phase and removed ≤5 hours after light offset. The time of lens application and removal varied daily in a pseudorandom manner that prevented entrainment to arousal and handling.

#### Maintenance of circadian entrainment following lens switch

To determine whether a previously-occluded eye could maintain circadian entrainment, some hamsters were subjected to a lens switching manipulation on week 10. 3 hamsters in the BB condition were switched to the BR condition, 10 hamsters in the BR condition had occluders shifted from the right to the left eye (BL), and 6 hamsters in the CR condition were switched to either a BR or BL treatment. This yielded the following treatment groups: CR→CR (n = 6), BB→BB (n = 2), BB→BR (n = 3), BR→BR (n = 15), BR→BL (n = 10), CR→BR (n = 2), and CR→BL (n = 4). Following this lens reassignment, the new lens assignments were maintained daily for thereafter. Entrainment of the locomotor activity rhythm to the light-dark cycle was monitored over the next 6 weeks (weeks 10–16).

#### Phase-shifting after lens switch

To determine whether a previously-occluded eye could mediate phase-resetting responses of the circadian system to light, the light-dark cycle was phase-advanced by 5 h in a single day on week 16 (lights on at 10:00, off at 12:00 CST). Locomotor activity was assessed in the home cage during the following weeks.

### Experiment 3 Procedure: Perceptual effects of monocular deprivation

#### Visual acuity tests

To assess acuity of the primary visual system, on week 15 hamsters from experiment 2 were challenged on week 15 with tasks that required them to identify and respond to a visual target (using procedures adapted from Finlay et al. [47]). For 2 days prior to behavioral testing, hamsters were habituated to an aliquot of unsalted sunflower seeds (David, Omaha, NE), which they readily consumed. All tests were performed in the home cage, during the 2 h light phase; lenses were in situ when tests were conducted. Experimenters were blind to treatment condition.

Behavioral tests of object recognition and motion detection [47] were performed in the following order: (1) CHEEK: a sunflower seed was held adjacent to the right cheek with a pair of stainless steel forceps. (2) NEAR: the seed was held in forceps approximately 2 cm in front of the nose. (3) HALF-CAGE: a hamster was placed at one end of the cage and the seed was held a half cage-length away. (4) FULL-CAGE: a hamster was placed at one end of the cage and the seed was held one full cage length away. (5) CLOCKWISE: the seed was held 2 cm in front of the hamster and rotated clockwise in a 360° circle around its head. (6) COUNTER-CLOCKWISE: the seed was held 2 cm in front of the hamster and rotated counter-clockwise in a 360° circle around its head. (7) OVERHEAD: the seed was held in front of a hamster's nose and rotated 90° vertically, stopping 2 cm above its head. Clear movement of the body (test 2–4) or head (tests 1, 5–7) in the direction of the seed was scored as a PASS; the absence of clear movement was scored as a FAIL.

### Experiment 4 Procedure: Effects of monocular deprivation on light-induced c-fos expression in the SCN

Hamsters from experiment 2 remained in the shifted 2L∶22D light-dark cycle and lensing treatments established on week 10 were continued for an additional 7 months. On week 38, occluder treatments ceased and all hamsters were transferred to DD. Hamsters were allowed 3 weeks to acclimate to DD and establish clear free-running rhythms in locomotor activity. On week 41, hamsters were fitted with an opaque occluded in the left or right eye and transferred (in their home cages) to a light-tight chamber 5 minutes prior to CT19 (circadian time 19; 7 circadian hours after the onset of locomotor activity). At CT19 hamsters were subjected to either a non-saturating 1 min light pulse (LP; 8.22 µW/cm^2^) or no light (dark pulse; DP). Mean light irradiance was measured at 6 points across the cage floor using a PR-650 SpectraScan Colorimeter (PhotoResearch: Chatsworth, CA).

This treatment yielded several final treatment groups based upon lensing history and whether the hamster received a light or dark pulse: BB→BB/LP-L (n = 1), BB→BB/LP-R (n = 1), BB→BR/DP-R (n = 2), BB→BR/DP-L (n = 1), BR→BL/LP-R (n = 3), BR→BL/LP-L (n = 5), BR→BR/DP-R (n = 1), BR→BR/DP-L (n = 1), BR→BR/LP-R (n = 7), BR→BR/LP-L (n = 7), CR→BL/LP-R (n = 2), CR→BL/LP-L (n = 4), CR→CR/LP-R (n = 4) and CR→CR/LP-L (n = 5).

For the purposes of analyses, an eye that received an LP (or DP) on week 41 was categorized based on its lensing history prior to and after week 10. Thus, an eye that received a LP (or DP) was either occluded up to week 10 then occluded after week 10, occluded→exposed, exposed→occluded, or exposed→exposed. For example, a BR→BL hamster receiving a LP in the right eye would be categorized as occluded-exposed; a BR→BL hamster→receiving a LP in the left eye would be exposed-occluded.

Pulses were delivered at CT19 and 90 minutes later hamsters were deeply anesthetized in the dark with sodium pentobarbital (Nembutal, 100 mg/kg, i.p.) and transcardially perfused with 200 ml 0.9% saline followed by 200 ml 4% paraformaldehyde in 0.1 M phosphate buffer (PBS). Brains were removed and postfixed in 4% paraformaldehyde for 24 h, then transferred to 30% sucrose in 0.1 M PBS at 5°C for 3–5 days. Brains were stored at −80°C until they were cut into 40 µm coronal sections on a cryostat. Every third section was set aside for immunohistochemical analyses. Sections were stored at 5°C in cryoprotectant (PBS with 30% sucrose, 30% ethylene glycol and 1% polyvinylpyrolidone).

#### Immunohistochemistry

c-Fos immunohistochemistry was performed according to established methods [48]. Briefly, free-floating sections were washed in PBS, cleared of endogenous peroxidases with hydrogen peroxide, and incubated in normal goat serum (NGS, Vector Labs), after which sections were incubated at 5°C for 72 h in PBS/triton-X detergent (PBS-X) containing 2% NGS and 1∶10,000 primary antibody for c-Fos (rabbit anti-mouse; CalBiochem). Sections were washed and then incubated for 90 min in PBS-X containing 2% NGS and 1∶500 secondary antibody (biotinylated goat anti-rabbit; Vector Labs). Sections were then washed, processed with avidin-biotin complex (Vector Labs), and visualized using the nickel-enhanced DAB system (Sigma). Sections were mounted (Superfrost slides, Fisher), air-dried, dehydrated in a series of ethanol dilutions, cleared (Histoclear, Fisher) and coverslipped (Permount fixative, Fisher).

#### Cell counting

Brain sections were photographed on an Eclipse 50i microscope (Nikon, Tokyo, Japan). SCN-containing sections were determined by comparing easily identifiable anatomical landmarks to those in a stereotaxic atlas of the Syrian hamster brain [49]; a minimum of 3 sections was identified for each brain (range = 3–5). Sections were photographed in color at 20× using a CCD camera (QImaging, Surrey, BC, Canada). Color (16 bit RGB) TIFF images (2048×1536 pixels) were generated using QCapture Pro (QImaging) and exported to Adobe Photoshop CS2 (Adobe Systems, San Jose, CA). Templates that best matched the anatomical landmarks of the SCN were superimposed onto these images [49]. Fos-immunoreactive (IR) cells inside the boundary of the SCN were hand-counted using ImageJ (NIH, Bethesda, MD) with the Cell Counter plugin by an experienced researcher blind to treatment condition. For each subject, the first section matching the template for bregma −0.9 mm was used in the analysis.

### Experiment 5 Procedure: Effects of monocular deprivation on retinohypothalamic projections

To determine whether early occlusion affects development of the RHT, a separate experiment was conducted in which hamsters were subjected to eye-specific lensing, followed by fluorescent cholera toxin-b subunit labeling of retinal ganglion cells projecting to the SCN.

Hamsters (n = 36), housed in DD≥3 days prior to birth, were weaned on postnatal day 16 and assigned to one of 4 treatment groups. Lensing began 1 day prior to the implementation of a 2L∶22D light-dark cycle on day 18. Nomenclature describing the treatment groups is identical to that used in experiment 2, although hamsters remained in their original condition throughout the experiment (i.e., there was no lens reassignment or shifts in the timing of the light-dark cycle). BB hamsters (n = 8) received opaque occluders over both eyes. BR (n = 9) and BL (n = 7) hamsters received occluders over the right and left eyes, respectively. CR hamsters (n = 9) received clear lenses over the right eye only. Additional no-lens (NL) controls (n = 7) did not receive lenses. As in experiments 1 and 2, lensing was performed daily before light onset and lenses were removed after light offset. Lenses were applied daily for 38 weeks. Locomotor activity was monitored continuously.

#### Tracers

Common monochromatic immunohistochemical tract-tracing methods (e.g., HRP) preclude identification of projections from separate retinas in the same animal [50]. Instead, the b subunit of cholera toxin (CTb), pre-conjugated to a fluorophore, was used. This toxin is effective in non-transsynaptic anterograde tract tracing [51]. The cholera toxin-b subunit employed in this study was conjugated to the fluorescent dyes Alexa Fluor 488 (CTb/AF-488; peak absorption 495 nm, peak emission 519 nm) and Alexa Fluor 594 (CTb/AF-594; peak absorption 590 nm, peak emission 617 nm), which appear green and red, respectively. Injecting CTb conjugated to a unique fluorophore into each eye allowed direct discrimination of projections from each eye separately within the same brain section.

#### CTb tracing

On week 38 each hamster was lightly anesthetized with isoflurane vapor and injected with CTb/AF-488 or CTb/AF-594 according to methods described in detail elsewhere [52]. Briefly, injections were delivered to the vitreous chamber of each eye, which was pierced with a blunted needle (26 g) attached to a 10 µl Hamilton syringe. To minimize leaking of injection solutions due to the high pressure of the eye vitreous humor, solutions were injected slowly (≥30 sec/10 µl), and the needle remained in the eye for 15 seconds after dispensing the solution. Substantial leakage occurred following needle withdrawal in 3 hamsters, which were excluded from all subsequent analyses. Tracers were diluted in 4% dimethylsulfoxide (DMSO) to achieve equimolar concentrations of each fluorophore. Each hamster received 10 µl of CTb/AF-488 (C34775, Molecular Probes, Eugene, OR) in one eye, and 10 µl of CTb/AF-594 (C22842, Molecular Probes) in the opposite eye. Fluorophores used in the left and right eyes were counterbalanced across all treatment groups.

Two days after injections, hamsters were deeply anesthetized with sodium pentobarbital (Nembutal, 100 mg/kg, i.p.) and transcardially perfused with 200 ml 0.9% saline followed by 200 ml 4% paraformaldehyde in 0.1 M PBS. Brains were removed and postfixed in the 4% paraformaldehyde for 24 hours, then transferred to 30% sucrose in 0.1 M PBS at 5°C for ≥3 days. Brains were stored at −80°C until sectioned at 40 µm on a cryostat. Every third section was mounted on Superfrost slides, air-dried, coverslipped with Permount, and stored in darkness until digital images were acquired.

#### Image acquisition and analysis

Brain sections were observed on a Nikon Eclipse 50i microscope fitted with a D-FL epi-fluorescence attachment and a 100 W mercury lamp. SCN-containing sections were determined by comparing easily identifiable anatomical landmarks to those in a stereotaxic atlas of the Syrian hamster brain [49]; a minimum of 3 sections was identified in each hamster (range = 3–5). Sections were photographed using both the 488 nm and 594 nm fluorescent filters at 20× using a CCD camera.

Grayscale (8 bit) TIFF images (2048×1536 pixels) were generated using QCapture Pro and exported to Adobe Photoshop CS2. Templates that best matched the anatomical landmarks of the SCN were superimposed onto these images [49]. The brightness level of all pixels outside of the boundaries of the SCN was set to 0 (black). The rostral-most matching SCN section was designated template 1 (T1); more caudal sections were designated T2 and T3, in order. This provided a coarse estimate of RHT innervation of the SCN in the rostral-caudal dimension without the need to section brains in the sagittal plane.

A quantification technique described by Muscat et al. [52] was used to assess density of retinal projections to the SCN. The mean brightness of an SCN-containing rectangle from an uninjected control hamster was used to quantify background fluorescence under each excitation wavelength. NIH ImageJ software (version 1.39, NIH, Bethesda, MD) was used to convert grayscale images to binary (black and white) images via the Adjust>Threshold command. Threshold was set at 30% above the background level, a value previously established as effective in discriminating fluorescently labeled projections from background in the hamster lateral geniculate nucleus [53]. All pixels above this value were recorded as “signal.” Signal pixels from T1, T2 and T3 sections were counted for each side of the SCN. Signal intensity for all lensed hamsters was then standardized by expressing the fluorescence signal value as a percentage of the mean fluorescence signal value generated by the NL group. This was performed separately for each emission wavelength to generate a standardized fluorescence index (SFI).

### Statistical analyses

Statistical analyses were performed using Statview (Version 5.0.1, SAS Institute, Cary, NC). Repeated measures ANOVA was used to compare patterns of entrainment in experiment 2. X^2^ tests and Fisher's Exact tests were used to compare performances on visual acuity tests in experiment 3. One-sample student's t tests were used to compare the periods of circadian activity rhythms in experiment 5. One-way factorial ANOVAs were used for all comparisons of Fos-IR cell counts and fluorescence measurements in experiments 4 and 5, respectively. Post-hoc pairwise comparisons were conducted where appropriate using or t-tests or Fisher's PLSD tests to protect against type 1 error introduced by multiple comparisons. Differences were considered significant if p<0.05.
